# Disassembly-based bill of materials data for consumer electronic products

**DOI:** 10.1038/s41597-020-0573-9

**Published:** 2020-07-30

**Authors:** Callie W. Babbitt, Hema Madaka, Shahana Althaf, Barbara Kasulaitis, Erinn G. Ryen

**Affiliations:** 1 Golisano Institute for Sustainability, Rochester Institute of Technology, Rochester, NY 14623 USA; 2 Finger Lakes Community College, Canandaigua, NY 14424 USA; 3 Wells College, Business Department, Aurora, NY 13026 USA

**Keywords:** Environmental impact, Sustainability, Technology

## Abstract

Consumer electronic products have a complex life cycle, characterized by environmental, social, and economic impacts and benefits associated with their manufacturing, use, and disposal at end-of-life. Accurately analysing these trade-offs and creating sustainable solutions requires data about the materials and components that make up these devices. Such information is rarely disclosed by manufacturers and only exists in the open literature in disparate case study format. This study presents a comprehensive database of bill of material (BOM) data describing the mass of major materials and components contained in 95 unique consumer electronic products. Data are generated by product disassembly and physical characterization and then validated against external benchmarks in the literature. The study also contributes a reproducible framework for organizing BOM data so that they can be expanded as new products enter the market. These data will benefit researchers studying all aspects of electronics and sustainability, including material scarcity, product design, environmental life cycle assessment, electronic waste policy, and environmental health and safety.

## Abstract (summary)

**Table:** 

Measurement(s)	consumer electronic product • bill of materials • material type
Technology Type(s)	Disassembly • weighing • characterization
Factor Type(s)	product category • product design
Sample Characteristic - Environment	manufactured product

Machine-accessible metadata file describing the reported data: 10.6084/m9.figshare.12513695

## Background & Summary

Consumer electronics have enabled a revolution in the way society accesses and shares information, education, and entertainment. But this transformation has come at a significant environmental cost. Satisfying society’s hunger for new technology has led to concerns about energy- and resource-intense product manufacturing^1^, declining product lifespans^2^, and waste management systems that create risks to human and environmental health^3^. A major driver of these environmental risks are the vast and complex array of materials that enable the functionality and appearance that consumers demand, including valuable metals such as gold, silver, and platinum; scarce resources such as cobalt and rare earth elements; hazardous materials including lead and mercury; and difficult-to-recycle materials like polymers containing halogenated flame retardants^4,5^.

The ability to research and create sustainable solutions for electronic products hinges on the availability of high-quality data that accurately capture the materials and components contained in these devices. The environmental research field has made significant progress in developing comprehensive life cycle inventory databases describing environmental impacts associated with general processes associated with material extraction and refining (e.g., ecoinvent, GaBi, and U.S. LCI databases) and processes specific to electronics manufacturing^6,7^. These tools are typically applied to specific case study electronic products^1,8^, which makes it challenging to generalize to other product types, designs, or time periods.

To analyse the environmental footprint of an electronic product of interest, a first step is to establish the profile and quantity of materials, components, and assemblies present in that product (often called a bill of materials or BOM). These data can then be paired with existing environmental databases to determine the life cycle impact of electronic material procurement, product manufacturing, use, and disposal. For example, data on the mass of printed circuit boards (PCBs) contained in a computer could be coupled with life cycle inventory data, typically reported on a per mass basis, and used to estimate the resource use and environmental impacts of manufacturing circuit boards. BOM data are also vital for assessing the economic profile of electronic waste intended for recycling, the dependence of new technology on scarce minerals, and the human health risks associated with product handling during disassembly and recycling.

Therefore, this study was carried out to collect, verify, and disseminate BOM data that describe the major materials and components contained in common consumer electronic products. The primary goal was creating a transparent database for a wide cross-section of technologies and time periods that could be used by other researchers studying sustainable solutions for consumer electronics. Thus, the study focused on empirical data, obtained by extensive product disassembly and physical material characterization and organized into a reproducible framework. Recognizing that consumer electronics will continue to evolve in the future, this data set can be updated following this framework as new products enter the market and as other researchers publish studies containing BOM data. To this end, the study also evaluated existing examples of BOM data available in the open literature, which were found to vary widely in quality and reproducibility. Select literature values were also included to supplement the empirical BOM data.

## Methods

This study estimated the average bill of materials for 25 common categories of consumer electronics products using a combination of empirical analysis via product disassembly and physical material identification and measurement and external validation via literature benchmarking. Product categories (Table 1) were selected for study based on high ownership rates in U.S. households and prevalence in the electronic waste stream^9^. Within the 25 product categories analysed, a total of 95 individual products were disassembled, spanning a wide array of model years, product designs, and functional attributes (Table 1). These products were primarily obtained opportunistically or by request from donation events and e-waste recycling firms, although some were purchased as used devices from online resellers.

**Table 1:** List of 25 product categories analyzed.

Product category	Data points from lab (products disassembled)	Years covered by lab data	Data points from literature	Years covered by literature data
Basic mobile phone	9	1998–2010	0	—
Blu-ray player	3	2006–2012	0	—
CRT monitor	0	—	6	1990*
CRT TV	0	—	3	1987*
Desktop – integrated	1	2011	0	—
Desktop – traditional	1	2009	9	1985–2010
Digital camcorder	1	1998	2	Unknown
Digital camera	8	2002–2010	2	Unknown
Drones	4	2013–2016	0	—
DVD player	3	2004–2005	4	Unknown
E-reader	2	2010–2014	2	2001–2010
Fitness tracker	6	2012–2014	0	—
Gaming console	3	2005–2006	3	Unknown
Laptop	16	1999–2011	0	—
LCD monitor	2	2006–2008	10	2009*
LCD TV	1	2009	12	2002–2008
LED TV	1	2016	1	2011
LED monitor	2	2014–2016	0	—
MP3 player	5	2004–2010	1	2009
Netbook	3	1998–2008	1	2009
Non-smart thermostat	2	2011–2015	0	—
Smart thermostat	2	2011–2015	0	—
Plasma TV	0	—	8	2002*
Printer	5	1999–2009	5	2001*
Smartphone	12	2004–2015	0	—
Tablet	2	2011–2014	1	2009
VCR	1	1990	6	1986–2002

*Denotes products from published sources that have incomplete or uncertain information regarding production date. The year stated is the best approximation by those studies or by these authors based on model details or other specifications given.

### Collecting lab-scale bill of materials data via disassembly

A standard disassembly procedure was designed based on examples of BOMs in the literature^10–23^ and followed to ensure consistent data collection across multiple researchers who contributed to the disassembly dataset. The process of disassembly started by recording the mass of the full product assembly. The full weight included product power cords if they were affixed to the product (as opposed to detachable). Subsequently, the product was disassembled to its major assemblies, which were assigned a unique number and description. The number and organization of unique assemblies varied by product, depending on the complexity of the product’s design and the logical way in which its internal components could be grouped.

**Fig. 1:**
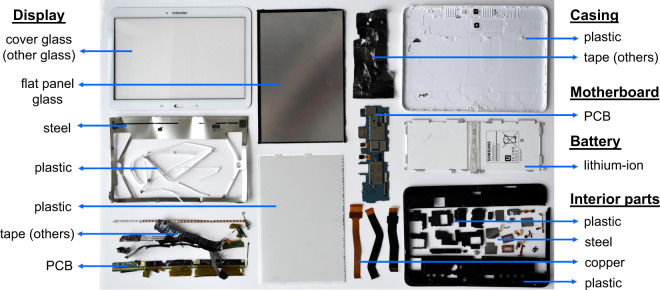
Example of product disassembly for a tablet (Samsung Galaxy Tab 4 SM-T530, 2014), illustrating the disaggregation achieved via lab disassembly and physical identification and measurement of representative assemblies (underlined terms), components, and materials.

For example, a tablet (Fig. 1) was disassembled into five assemblies: battery (lithium-ion battery cells and associated connectors), motherboard (includes PCB), display (includes flat panel glass, cover glass, display bezel, PCBs, plastic films, and other connectors), casing (back cover including camera lens), and interior parts (includes small PCBs and miscellaneous metal and plastic parts). Screws and other small parts from the same major assembly were grouped and weighed together. On the other hand, smartphones were observed to have more streamlined designs that could be described within two assemblies: main body (includes motherboard, interior parts, and battery) and display (includes flat panel glass, cover glass, plastic films, bezel, and other connectors).

Each of the major assemblies was weighed and then disassembled as far as possible with physical separation techniques (hand and power tools). Ideally, disassembly led to parts that were comprised of a single type of material, which could be classified as copper, steel, aluminum, other metals (typically magnesium), glass, or plastics (Fig. 1). These classifications were made based on visual inspection, physical properties, manufacturer labels, and recycling codes. Metal identification was verified using a Delta handheld XRF analyzer (Model DP-2000CC, >99% accuracy for Fe and Al and 95% accuracy for Mg, determined by repeated measurements using a reference alloy with known composition). For example, metals were first tested for ferrous content using a magnet. If magnetic properties were not observed, the metal is assumed to be either stainless steel or aluminum, and then verified with XRF. Copper was primarily identified based on visual inspection (e.g., copper wiring), and magnesium was identified using manufacturer label (parts stamped with a label indicating “Mg”) and verified with XRF. The small fraction of material that could not be classified into these material types, including paper films, rubber, adhesives, or epoxies, was classified as “others.”

The disassembly process also resulted in components that were composites of multiple materials that were partially or totally inseparable by physical means alone. For example LCD display modules could be separated to the point where some materials were individually identifiable, such as the display frame (plastic or steel), the polarizer and optical films (plastic and paper/others), and in some cases a tempered glass cover (other glass). However, the flat panel glass itself is a composite made up of multiple layers and materials, including a glass substrate, liquid crystal layer, transparent electrode, and other films, which were not further separable. Components like lithium-ion batteries and printed circuit boards (PCBs) themselves contain many of the same materials reported in the BOM, such as aluminium, copper, steel, and plastic, as well as other elements, including gold, silver, cobalt and lithium, all of which would only be separable by chemical or thermal techniques that are outside the scope of this study. Thus, the total mass of the component, at a point where it was no longer separable by physical disassembly, was recorded and reported in the BOM. As a result, the total amounts of individual materials in the BOM only represent the content of those materials present in a distinct, separable form in the product. The reported mass of components may include additional amounts of those materials and other elements that are not reported here but that can be estimated by connecting this study’s data with literature that has reported elemental concentrations, such as the mass of precious metals contained in PCBs^24^ or the mass of indium contained in flat panel display glass^25^.

All of the above mentioned mass measurements were collected using three balances, which were selected according to the size and weight of the part or material being weighed: 50 kg capacity (Acculab bench scale, model SVI-50C with 5 g resolution), 30 kg capacity (Measuretek high precision counting scale, model EHC-CF-30, with 1 g resolution), and 200 g capacity (Fisher Science compact balance, model CLF201, with 0.1 g resolution). The final mass of all the assemblies, and their respective sub-assemblies, components, and materials were compiled into a BOM for each product.

### Collecting literature bill of materials data

Because some BOM data already exist in the open literature, available sources were collected and assessed for potential to include in the BOM datasets (Table 1). One challenge was that literature BOM data are often presented in varied formats, according to the purpose of the study for which the material data were collected. Therefore, selection of literature sources^10–23^ to include alongside empirical data was based on three parameters: traceability, level of detail, and category consistency. Traceability refers to the degree of transparency in an article’s methodology with respect to how product disassembly and BOM construction were carried out, or in other words, the ability to trace reported material composition data back to methods as they were explained in the paper. Level of detail refers to the degree of disaggregation in the reported data, ranging from studies that only report final cumulative mass percent (low detail) to detailed component-level disassembly data (high detail). Finally, category consistency refers to the degree of similarity between the material categories considered in this study and those reported by the published sources. For example, some literature BOMs report “metals” content as opposed to breaking this down into specific types of metals (steel, aluminum, copper). Each parameter is rated as high, medium, or low depending on the published source (Table 2).

**Table 2:** Assessment of literature BOM data sources.

Reference	Traceability	Level of detail	Category consistency
AEHA reported in Oguchi *et al*.^19^	low	low	low
California Department of Toxic Substances control report^23^	medium	medium	medium
Chancerel and Rotter^20^	medium	medium	medium
Hikwama^18^	high	high	high
Huisman^14^ via Huisman *et al*.^13^	medium	medium	high
Huisman *et al*.^13^	medium	medium	high
JEITA reported in Oguchi *et al*.^19^	low	low	low
JOGMEC reported Oguchi *et al*.^19^	low	low	low
Kozak and Keoleian^15^	high	high	high
Lee and Hsi^11^	medium	medium	high
MoE reported in Oguchi *et al*.^19^	low	low	low
Oguchi *et al*.^19^	low	low	low
Peeters *et al*.^10^	low	low	medium
Socolof *et al*.^17^	medium	medium	high
Streicher Porte *et al*.^21^	medium	medium	medium
Stobbe^16^	high	high	high
Teehan and Kandlikar^12^	high	high	high
Tohoku Bureau of ETI via Oguchi *et al*.^19^	low	low	low
Townsend *et al*.^22^	low	low	medium

Based on this assessment, one of three scenarios was typically observed, which determined how the literature data were treated and whether they were ultimately included in the final average BOM values (Online-only Table 1):

Scenario 1: Literature reported a transparent product disassembly methodology, fully detailed bill of materials with major component assemblies and subassemblies. For example, Teehan and Kandlikar^12^ manually disassembled fourteen different products following a methodology similar to that used in this work. Complete BOM were reported, including information on model number and year. In cases like this, the literature data could be directly aligned to the primary BOM data sets with no or minimal adjustments (e.g., aggregating material compositions at a product level).

Scenario 2: Literature provided a transparent product disassembly methodology, but the reported BOM are only partially detailed or reported in a different format, and thus required processing for consistency with the primary disassembly data. For example, a study by the California Department of Toxic Substances^23^ also used direct disassembly of 19 products to find composition of major component assemblies. Based on the goals of that study, only the mass of major components (PCBs, LCD panels, and fluorescent bulbs within LCD lighting) were detailed, and no distinctions were made between types of metals present in the products. To align these data with the BOM dataset, some minor processing was required, such as disaggregating the “total metal” category into specific metal categories (aluminum, copper, steel and other metals) according to the percentages observed for similar products in the primary disassembly data. This assumption was based on empirical observation of consistent relative contributions of specific metals within most product categories.

Scenario 3: In studies where reporting a BOM is not the primary goal of the research, material data may be presented without a full explanation of methods or compositional breakdown. For example, Oguchi *et al*.^19^ provided a comprehensive study on the characteristics of end-of-life electronics as a potential source for metals recovery. Because the study goal was quantifying the metal content present in electronic products, there was less focus on other materials, such as plastics or glass. As a result, the published material data do not sum to 100% of the product mass. In these cases, the partial data are listed in the BOM datasets with the missing mass percentage composition assigned to the “other” category. Because these data have a fundamentally different structure, they cannot be compared directly to the primary disassembly results and are not included in final average mass compositions reported.

Subsequent to these determinations, literature values that reflected scenarios one and two above were included in determining average material compositions for each product category as follows

$${\rm{Average}}\,{\rm{material}}\,{\rm{composition}}=\frac{{\sum }_{i=1}^{n}P{D}_{i}+{\sum }_{j=1}^{N}L{D}_{j}}{n+N}$$

where *PD*_*i*_ is the material composition for each product “*i*” disassembled in the lab, and *n* is the number of products disassembled in that product category. *LD*_*j*_ is the material composition for each product “*j*” taken from literature, and *N* is the number of products taken from literature for the product category. The final calculated averages are shown in the data record described below and the summary BOM (Online-only Table 1).

## Data Records

The BOM datasets are available at figshare^26^. These data records are compiled in two Excel workbooks containing BOM data collected and organized at different levels of aggregation, corresponding to the ways in which researchers might need to access this information. First, the “Disassembly Detail” workbook provides resolved material and component data at the level of each major assembly and subassembly. Each worksheet represents a single product category, and most categories contain detailed data for multiple product samples (Table 1). An example of these results is provided here (Table 3) for the tablet pictured in Fig. 1, demonstrating how the disassembly and material identification processes were translated into an assembly-level BOM.

**Table 3:** Example of assembly-level BOM data for a tablet (Samsung Galaxy Tab 4 SM-T530, 2014), illustrating how data are presented in the “Disassembly Detail” workbook. Mass data are in grams.

Material	Material and mass (g) breakdown by component:					Total material mass
	Casing	Display	Battery	Interior parts	Motherboard	
Aluminum						
Copper				2.2		2.2
Steel		20	0.3	6.1		26.4
Plastic	65	43.5		41.3		149.8
Li-ion battery			125			125
PCB		7.3		2.4	26.2	35.9
Flat panel glass		60				60
CRT glass						
Other glass		90				90
Other metals						
Others	1	1.4		0.2		2.6
Total component mass	66	222.2	125.3	52.2	26.2	491.9

Second, the “Product Bill of Materials” workbook provides total mass and mass percent of each separable material and component for all products studied and a mean, maximum, and minimum mass (g) and mass percent (%) for each product category calculated using the lab data points. The workbook also contains literature values, which were collected, evaluated, and processed according to the methods section reported above. If available, assembly-level literature BOM data are included, however, it was more common to find published data presented as mass percentages for the product as a whole. The qualitative analysis of data from published literature is indicated next to each data point. An example of these results is provided here (Table 4) for the tablet category, which included two disassembly-based data points and one high-quality literature data point, all of which were reflected in the product category average BOM. A summary table containing the product-level average BOM values is also provided as Online-only Table 1.

**Table 4:** Example of product-level BOM data for the tablet category, illustrating how data are presented in the Product Bill of Materials workbook.

Material categories	This study				Literature^12^		Average mass %
	Samsung (2011)		Samsung (2014)		Apple iPad (2009)		
	Mass (g)	Mass %	Mass (g)	Mass %	Mass (g)	Mass %	
Aluminum	45.0	8.0	—	—	137	20.0	9.3
Copper	3.9	0.7	2.2	0.45	1.1	0.16	0.4
Steel	28.1	5.0	26.4	5.4	12.5	1.8	4.1
Plastic	127	22.7	150	30.5	36.4	5.3	19.5
Li-ion battery	135	24.1	125	25.4	129	18.8	22.8
PCB	54.4	9.7	35.9	7.3	20.1	2.9	6.6
Flat panel glass	55.0	9.8	60.0	12.2	154	22.5	14.8
CRT glass							
Other glass	110	19.6	90.0	18.3	188	27.4	21.8
Other metals	—	—	—	—	—	—	—
Others	1.5	0.27	2.6	0.53	7.5	1.1	0.6
Total mass (g)	560		492		686		

Cells left blank indicate that the specified material is not applicable to this product. Cells with “—” indicate that the specified material was not detected by physical separation of the product.

In Table 4, Online-only Table 1, and in the Product Bill of Materials workbook, ‘zero’ values for specific materials could result for different reasons, which were conveyed by different cell formatting. Cells left blank indicate that a material is ‘zero’ because it is not applicable to the product. For example, CRT glass is found in CRT TVs and monitors, and lithium-ion batteries are found in mobile products, but these components are not expected to be present in other product categories. Cells containing “—” indicate that a material was not detected by the stated disassembly and material identification process, but we cannot rule out the potential that it is present in the product within a composite component or in a form or concentration not detected by these methods (e.g., as an additive, alloying element, tramp element, or contaminant, etc.). For example, the total reported mass of a PCB (component) would likely include individual materials present in that component (including, for example, aluminium and copper) that are not detectable or separable by physical disassembly alone. However, stated values for each material reflect only the mass of that material when it is separable and quantifiable by physical disassembly, and do not include additional quantities potentially contained in non-separable components (i.e., materials are not double counted).

## Technical Validation

Data were validated using quality controls within the study (internal validation) and by best available benchmarks to product market information and literature values (external validation).

One aspect of validation was evaluating if the disassembly and material identification methods were implemented without errors or variations that may introduce uncertainty to the results. In part, such uncertainty was mitigated by using a standard procedure and instruments (balances, XRF) with sufficient resolution and accuracy for the size and type of measurements made (see instrumental specifications in the Methods section). This uncertainty was also assessed by identifying data points that could be re-evaluated using multiple estimations. Specifically, the total product mass was determined prior to disassembly (for most products) and then re-estimated by summing the masses of individual materials and components after disassembly. Variability between these two estimates would point to small parts or materials lost to disassembly or inaccuracies in instrumentation. Data in the “Uncertainty Analysis” workbook, also posted to the figshare repository, demonstrates that the percent difference between these two mass measurements was about 0.5% on average, with a maximum of 2.5% for a single product.

To validate these measurements against external references, product mass estimated as the post-disassembly sum of material and components was also compared to reported weights from manufacturers, where such information could be obtained for the same make, model, and year product as studied in the lab. These comparisons showed about 1% difference on average, with a maximum of about 10% difference between values. It appeared that the few products with greater differences may be due to exclusion of the power cord in the BOM mass. Because many of the products disassembled were obtained from the e-waste stream, peripheral items like cords were not consistently available, and so they were excluded unless they were affixed to the product. Thus, final mass values may underestimate total mass in cases where a detachable power cord is sold with a product but not captured in the BOM. Other small discrepancies may represent uncertainty associated with disassembling products that may have been customized or upgraded after purchase, which would influence the final weight. However, these cases were few, and the majority of mass estimates were very close to available product specifications, providing additional confidence that products disassembled in the lab represent realistic models of the product, as described by the brand or third party verified websites.

The above approaches to validation are limited to total product mass, as no comparable internal measurement was available for repeating estimates on individual material identification or mass. However, disassembly and material identification data could be validated against literature sources if a comparable product were available. From the dataset, two products were identified as being very similar to both our lab data and a high quality literature study^12^: an Amazon Kindle from 2010 and an Apple iPod Touch 8 Gb from 2008/2009. The products were close but not exact matches, as the Kindle described in the literature was a third generation model of the original design and the one disassembled was a first iteration of a slightly altered design. The iPods were identical in make and model, but were potentially manufactured in different years. The lab study identified the iPod to be from 2008, based on the date stamped on the case; the literature only reported it as ‘circa 2009’. However, these are the most similar options available to provide BOM validation.

Side-by-side comparisons of the BOMs for both products are included in the Uncertainty Analysis workbook. For the Kindle, total product mass reported in both BOMs differed by only 1.7 g (0.8%) while mass reported for specific assemblies varied by +/−5 g or less, typically due to small differences in how parts were assigned (e.g., assigning screws to ‘interior parts’ vs. ‘casing’ assemblies). The mass contribution by specific materials and components were also highly consistent, barring one exception, where this study found an approximately 20 g internal backplate to be steel (verified by magnetic properties and XRF) and the literature study assigned it as aluminum based on the lack of magnetic properties. The discrepancy is likely due to small design or manufacturing differences between the two models. For the iPod, total product mass reported in both BOMs differed by only 1.6 g (1.4%) while mass reported for assemblies was +/−1 g or less. The mass contribution by specific materials was extremely close between the two BOMs, with the biggest variability (3.8 g) stemming from this study assigning the plastic frame surrounding the flat panel screen to the “plastics” category, while the literature study included it in the LCD display category.

While this detailed level of comparison was not possible for all products, as no other model and year overlap was found, the two examples provided show high agreement, indicating that the methods of disassembly and material identification were robust. However, it should be noted that the applicability of reported BOM findings to studies involving current electronic products will depend on the similarity of product designs and the extent to which technology has evolved over time. Many of the products included in this data set are older models, currently being discarded. As such, they are good representations of materials and components now found in the e-waste stream, but not necessarily generalizable to new technologies being manufactured and sold currently. For relatively well-established technologies, the overall material composition has been shown to remain relatively constant over time, particularly once a specific design and form factor is established in the market^8^. For emerging technologies, materials are not yet well understood and will require additional study and BOM characterization. However, the framework for disassembly, material identification, and measurement presented here can be adapted to collect additional data for new products as they become available.

## Online-only Table

**Online Table 1:** Average bill of materials for consumer electronic products.

Product category	Average bill of materials (mass percent)											Total product mass (grams)		
	Aluminum	Copper	Steel	Plastic	Li-ion battery	PCB	Flat panel glass	CRT glass	Other glass	Other metals	Others	Average	Maximum	Minimum
Basic mobile phone	5.6	2.1	1.0	34.0	24.9	18.2	8.1		—	—	6.0	117	173	95
Blu-ray player	0.2	3.5	59.8	17.5		17.6			0.4	—	1.0	3,150	4,460	983
CRT monitor	1.0	4.2	9.3	16.0		7.5		59.3	—	1.6	1.2	15,050	21,160	11,690
CRT TV	0.4	3.4	5.4	16.9		5.7		64.2	—	1.7	2.4	34,094	41,517	26,671
DVD player	1.1	4.1	48.6	33.2		12.8			—	—	0.2	3,692	5,161	2,338
Traditional / integrated desktop	8.7	3.9	52.2	20.9		9.9	1.0		1.2	0.9	1.2	8,795	12,320	2,971
Digital camera / camcorder	12.0	1.3	15.5	40.6	2.4	15.6	4.1		1.7	—	6.9	214	848	111
Drone	0.8	2.8	12.0	57.8	7.0	17.0	1.0		—	—	1.7	886	1,858	81
E-reader	6.1	0.4	4.6	32.1	20.7	12.1	16.2		5.3	2.2	0.3	323	515	222
Fitness tracker	3.2	1.2	19.4	18.9	7.8	6.5	3.2		—	—	39.8	25	41	16
Gaming console	9.1	2.2	31.3	34.5	0.10	16.6			—	0.04	6.2	2,719	4,228	1,178
Laptop	15.4	1.8	11.5	28.3	14	12.4	8.2		—	5.8	2.5	2,706	3,751	1,745
LCD monitor	6.2	5.3	35.8	28.4		6.2	17.9		—	—	0.2	4,711	6,893	2,829
LCD TV	2.5	0.9	42.6	28.1		5.8	12.9		—	4.7	2.5	20,779	29,983	14,406
LED monitor / LED TV	13.6	0.02	27.9	39.8		4.1	14.2		—	—	0.3	5,167	9,277	3,042
MP3 player	25.5	0.7	13.8	15.4	11.6	14.4	7.7		9.4	—	1.5	93	128	52
Netbook	10.0	1.4	5.5	29.1	17.5	16.1	11.3		2.2	4.3	2.7	1,087	1,322	896
Plasma TV	11.1	0.5	32.5	29.8		5.9	11.7		1.5	1.9	5.2	43,479	65,358	35,616
Printer	0.2	0.6	30.0	60.8		3.1	0.1		3.8	—	1.4	6,138	8,329	1,801
Smartphone	9.9	1.2	6.3	23.7	22.6	14.0	6.7		8.7	2.5	4.1	135	180	91
Smart thermostat	3.7	0.4	14.0	55.7	3.3	16.7	6.0		—	—	0.1	204	333	143
Tablet	9.3	0.4	4.1	19.5	22.8	6.6	14.8		21.8	—	0.6	579	686	492
VCR	—	1.5	64.6	18.6		15.3			—	—	—	3,861	N/A	N/A

Note:

Averages include disassembly-based lab data and high-quality literature data.

Cells left blank indicate that a material or component is not applicable to the product. For example, CRT glass is only found in CRT TVs and monitors and is not expected in other product categories.

Cells containing “—” indicate that a material was not directly detected or quantified, although it may be present in the product. For example, the total reported mass of a PCB (component) would likely include individual materials present in that component (aluminum, copper, gold) that are not detectable or separable by physical disassembly alone. Likewise, stated values for each individual material reflect only the mass of that material when it is separable, identifiable, and quantifiable by physical disassembly and not additional amounts of that material that may be contained in composite components (i.e., materials are not double counted).
